# A Boswellic Acid-Containing Extract Ameliorates Schistosomiasis Liver Granuloma and Fibrosis through Regulating NF-κB Signaling in Mice

**DOI:** 10.1371/journal.pone.0100129

**Published:** 2014-06-18

**Authors:** Miao Liu, Qingsi Wu, Peng Chen, Berthold Büchele, Maohong Bian, Shengjian Dong, Dake Huang, Cuiping Ren, Yuxia Zhang, Xin Hou, Thomas Simmet, Jijia Shen

**Affiliations:** 1 Department of Microbiology and Parasitology, Anhui Medical University, Hefei, Anhui, People’s Republic of China; 2 Anhui Provincial Laboratory of Microbiology and Parasitology, Anhui Medical University, Hefei, Anhui, People’s Republic of China; 3 College of Clinical Medical Sciences, Anhui Medical University, Hefei, Anhui, People’s Republic of China; 4 Institute of Pharmacology of Natural Products & Clinical Pharmacology, Ulm University, Ulm, Germany; 5 Department of Blood Transfusion, The First Affiliated Hospital, Anhui Medical University, Hefei, Anhui, People’s Republic of China; 6 Department of Clinical Laboratory, Hefei Second People’s Hospital, Hefei, Anhui, People’s Republic of China; Agency for Science, Technology and Research - Singapore Immunology Network, Singapore

## Abstract

Boswellic acid (BA)-containing extracts such as BSE have anti-inflammatory and immunomodulatory activity. In chronic schistosomiasis, the hepatic granuloma and fibrosis induced by egg deposition in the liver is the most serious pathological manifestations. However, little is known regarding the role of BAs in *Schistosoma japonicum* (*S. japonicum*) egg-induced liver granuloma and fibrosis. In order to investigate the effect of a water-soluble complex preparation of BSE, BSE-CD, on *S. japonicum* egg-induced liver pathology, liver granuloma and fibrosis were induced by infecting C57BL/6 mice with 18–22 cercariae of *S. japonicum*. *S. japonicum* cercariae infected mice were injected with BSE-CD at the onset of egg granuloma formation (early phase BSE-CD treatment after 4 weeks infection) or after the formation of liver fibrosis (late phase BSE-CD treatment after 7 weeks infection). Our data show that treatment of infected mice with BSE-CD significantly reduced both the extent of hepatic granuloma and fibrosis. Consistent with an inhibition of NF-κB signaling as evidenced by reduced IκB kinase (IKK) activation, the mRNA expression of VEGF (vascular endothelial growth factor, VEGF), TNF-α (tumor necrosis factor-alpha TNF-α) and MCP-1 (monocyte chemotactic protein 1, MCP-1) was decreased. Moreover, immunohistochemical analysis (IHC) revealed that the content of α-SMA in liver tissue of BSE-CD treated mice was dramatically decreased. Our findings suggest that BSE-CD treatment attenuates *S. japonicum* egg-induced hepatic granulomas and fibrosis, at least partly due to reduced NF-κB signaling and the subsequently decreased expression of VEGF, TNF-α, and MCP-1. Suppression of the activation of hepatic stellate cells (HSC) may also be involved in the therapeutic efficacy of BSE-CD.

## Introduction

Schistosomiasis is an important disease among the neglected tropical diseases. It is caused by several species of the genus schistosoma and remains a serious public health problem that affects more than 200 million people in 74 countries [1]–[2]. The three important species affecting humans are *Schistosoma haematobium*, *Schistosoma japonicum* and *Schistosoma mansoni*. *S. japonicum* is mainly prevalent in China. By the end of 2012, the total number of schistosomiasis japonica cases were estimated to be 240597, the total number of advanced schistosomiasis cases were estimated to be 30396. Schistosomiasis mainly was endemic in 7 provinces in the south of China, including Hubei, Hunan, Jiangxi, Anhui, Jiangsu, Yunnan, Guangxi, and Sichuan [3]. The total infection rate in fishermen was 40.4% in two fisherman villages in Yueyang County of Hunan Province, 58.6% schistosomiasis had hepatic fibrosis [4]. 66.2% schistosomiasis had hepatic fibrosis by Type-B ultrasonic examining the status of hepatic fibrosis from 447 chronic schistosomiasis patients in Tongling County of Anhui Province [5]. In schistosomiasis, the adult worms grow inside the mesenteric and portal vein system of the human body where they are laying eggs. The eggs become trapped mainly in the liver and gut, where they induce a vigorous granulomatous response. Subsequently, fibrosis and portal hypertension may develop, which are responsible for morbidity and the potentially fatal outcome in infected individuals. Thus, much of the clinical manifestation of schistosomiasis is ascribed to the egg-induced granulomatous inflammatory response and the associated fibrosis [6]. The latter is a serious consequence of *S. japonicum* infection that involves remodeling of the extracellular matrix (ECM) and excessive deposition of collagen, primarily by hepatic stellate cells (HSCs) [7]. Effective medical interventions to control and treat granuloma and fibrosis in schistosomiasis are lacking so far.

NF-κB is an important transcription factor involved in the expression of a variety of inflammatory genes in response to pathogens and cytokines [8]. It has been shown that activation of the NF-κB signaling pathway is closely associated with the development of granuloma and hepatic fibrosis [9]–[10]. Both Th1 and Th2 cytokines determine the hepatic granuloma size [11]–[12]. Particularly chemokines, such as MCP-1, contribute to the formation of hepatic granuloma in schistosomiasis. Accordingly, injection of anti-MCP-1 or anti-MCP-1α antibodies can inhibit granuloma formation [13]. Besides, VEGF is an important signaling protein involved in both vasculogenesis and angiogenesis. To that effect, previous studies have demonstrated that VEGF also plays an important role in promoting granuloma formation and fibrosis in experimental schistosomiasis [14]–[15].

Extracts of the oleogum resin from *Boswellia serrata* (BSE) containing a mixture of boswellic acids (BAs) have been confirmed to regulate inflammation and immune responses [16]–[17]. Likewise, recent studies have demonstrated that boswellic acid extracts attenuate pulmonary fibrosis and colonic fibrosis in rats [18]–[19]. Thus, these studies suggest that BSE might be valuable for the treatment of fibrosis associated with diverse clinical diseases. We have previously shown that water-soluble BSE-CD could attenuate *S. japonicum* egg-induced hepatic granuloma [20]. This prompted us to address in greater detail the role and mechanism of boswellic acids in *S. japonicum* egg-induced liver granuloma and the subsequent development of hepatic fibrosis.

The aim of our present work was to investigate whether the BA-containing extract (BSE) administered as a special cyclodextrin complex (CD), could attenuate *S. japonicum* egg-induced liver granuloma and fibrosis by a mechanism involving the inhibition of the NF-κB signaling pathway and the expression of selected NF-κB-dependent genes.

## Materials and Methods

### Ethics Statement

All mice were maintained in a specific pathogen-free microenvironment, and received care in compliance with the guidelines outlined in the *Guide for the Care and Use of Laboratory Animals*. All work was conducted with the approval of the Anhui Experimental Animal Training Base. The permit license number is LLSC2012-001.

### Parasites and Animals

*Oncomelania hupensis* snails, infected with the Chinese strain of *S. japonicum* were provided by the Jiangxi Provincial Institute of Parasitic Diseases. Six weeks old female C57BL/6 mice, weighing 18–20 g, were purchased from the Experimental Animal Center of Chinese Science Academy. They were housed in a specific pathogen-free microenvironment in the animal facility, and received care in compliance with the guidelines for the Care and Use of Laboratory Animals. All work was approved by the Anhui Experimental Animal Training Base.

### Infection of Mice with *S. japonicum*

Cercariae were shed in a beaker after exposing thirty infected *Oncomelania* to sunlight for 4 h (25–28°C). Mice were infected percutaneously with 20±2 *S. japonicum* cercariae.

### Treatment of Mice with BSE-CD

The German drug authorities (BfArM) had previously approved BSE for testing in clinical studies. The extract composition of major BA derivatives is as follows: 1. α-boswellic acid (138 mg/g), 2. acetyl-α-boswellic acid (34 mg/g), 3. β-boswellic acid (192 mg/g), 4. acetyl-β-boswellic acid (100 mg/g), 5. 9,11-dehydro-α-boswellic acid (2 mg/g), 6. acetyl-9,11-dehydro-α-boswellic acid (0.6 mg/g), 7. 9,11-dehydro-β-boswellic acid (8 mg/g, 8. acetyl-9,11-dehydro-β-boswellic acid (6 mg/g), 9. lupeolic acid (26 mg/g), 10. acetyl-lupeolic acid (12 mg/g), 11. 11-keto-β-boswellic acid (66 mg/g), 12. acetyl-11-keto-β-boswellic acid (38 mg/g).

To allow the application of lipophilic BAs in aqueous solutions, BSE was complexed with cyclodextrin (CD) yielding BSE-CD ready for *in vivo* applications. For early phase BSE-CD treatment, the animals were divided into 5 groups: BSE-CD (280 mg/kg), BSE-CD (140 mg/kg), cyclodextrin (280 mg/kg), infected mice and normal mice, every group had 8 mice. Mice were injected intraperitoneally with BSE-CD (280 mg/kg) or BSE-CD (140 mg/kg) every day for 21 days of successive administration starting at 4 weeks after infection. In analogy, control mice were given intraperitoneally injections of cyclodextrin (280 mg/kg) for 21 days of successive administration starting at 4 weeks after infection. Another group of infected mice did not receive any treatment. Mice were sacrificed 24 h after the last administration. For late phase BSE-CD treatment, the animals were divided into 5 groups: BSE-CD (280 mg/kg), BSE-CD (140 mg/kg), cyclodextrin (280 mg/kg), infected mice and normal mice, every group had 8 mice. Mice were orally administered praziquantel on week 6 to eliminate the adult worms followed by injection with BSE-CD or cyclodextrin control from week 7 until week 10.

### Histology and Immunohistochemistry of Liver Sections

Livers were excised from sacrificed mice, instantly fixed in 10% formalin in PBS and embedded in paraffin. Then tissue sections (4 µm) were stained with hematoxylin and eosin (H&E) or with Masson trichrome to examine the size of the granulomas and the extent of hepatic fibrosis, respectively. Immunostaining for phosphor-IκB kinase (IKK) was performed using a monoclonal phosphor-IKK primary Ab (Cell Signaling Technology, Beverly, MA, USA); immunostaining for α-smooth muscle actin (α-SMA) was performed using polyclonal α-SMA primary antibody (Bioss Inc., Bejing, China). Immunohistochemical analysis was performed with the Powervision Two-Step detection system (Zhongshan Biotechnology Co., Beijing, China). Six to ten microphotographs per mouse liver were recorded using an inverted microscope (Nikon 80I, Japan). Quantitative and qualitative changes were analyzed by means of morphometric software (Image-Pro Plus software). At least three non-continuous tissue sections were measured, and the mean values obtained from eight mice were used for statistical analysis.

### Quantitative PCR

Total RNA of fresh liver tissue was isolated with Trizol Reagent (Invitrogen, Carlsbad, CA, USA) and transcribed into cDNA. Quantitaive PCR was performed using an ABI-Prism StepOne Plus Sequence Detector Systems (Applied Biosystems) and SYBR Premix Ex Taq (Takara Dalian, China). The primers used in this experiment are listed in Table S1. For normalization, we used the housekeeping gene encoding β-actin. Gene expression values were then calculated based on the ΔΔCt method [21]. The mean of the respective cytokines in uninfected mice was used as a calibrator. The relative quantity of target cytokine mRNA was expressed as 2^−ΔΔCt^.

### Electrophoretic Mobility Shift Assay (EMSA)

For electrophoretic mobility shift assays (EMSA), we isolated nuclear proteins from the liver samples according to the manufacturer’s instruction; protein concentrations were determined using a BCA assay kit (Viagene Biotech, Ningbo, China). The binding of NF-κB to consensus oligonucleotides was analyzed using an EMSA kit (Viagene Biotech, Ningbo, China) according to the manufacturer’s instruction. The sequences of the oligonucleotides used for the EMSA are 5′-AGTTGAGGGGACTTTCCCAGGC-3′ served as the probe.

### Statistical Analysis

The results were analyzed by the Student’s *t*-test or analysis of variance where appropriate. All data were shown as mean ± standard error of the mean (SEM). *P* value <0.05 was considered to be statistically significant.

## Results

### Early Phase BSE-CD Treatment Suppresses *S. japonicum* Egg-induced Liver Granuloma Formation

**Figure 1:**
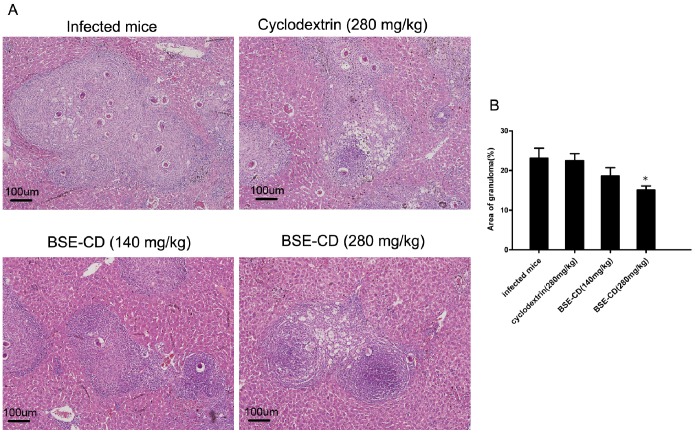
Effect of early phase BSE-CD treatment on hepatic granulomatous inflammation in mice infected with *S. japonicum*. Mice were infected with 20±2 cercariae, Treatment with BSE-CD or placebo from week 5 to week 7. Mice were killed, and liver tissues were fixed by formalin and stained with H&E. (A) Liver tissues stained with H&E. (B) Hepatic egg granuloma size was measured by computer-assisted histomorphometric analysis. All data are expressed as mean ± SEM (n = 8 for each group). *P<0.05 *versus* corresponding infected mice or the cyclodextrin (280 mg/kg) group.

To investigate the effect of BSE-CD on *S. japonicum* egg-induced liver granuloma, mice were intraperitoneally injected with BSE-CD or CD control once a day for a total of 21 days commencing 4 weeks after infection. Then mice were sacrificed, To investigate whether the above differences in liver granuloma size are due to dissimilar worm pairs, total worms, and total parasite eggs in the livers, we measured worm pairs, total worms, and total parasite eggs in the livers. Worm pairs, total worms, and total parasite eggs in the livers were similar between BSE-CD treatment groups and the control groups (Table S2)), liver tissues were fixed and stained with H&E. Strikingly, compared with control mice, early liver granulomas were attenuated in the group treated with 280 mg/kg BSE-CD. Likewise, in this group, H&E staining also showed a regression of the granulomatous inflammatory reaction compared with control mice (Fig. 1).

### BSE-CD Treatment Markedly Suppresses the NF-κB Signaling Pathway

**Figure 2:**
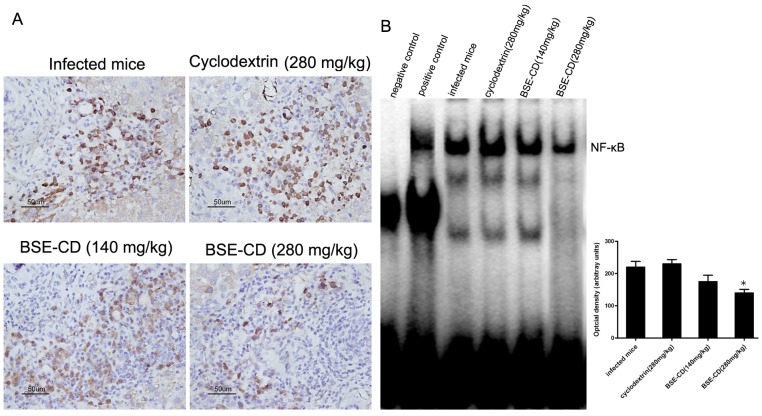
Effects of BSE-CD treatment on the NF-κB pathway activation in the liver tissue of mice infected *with S. japonicum*. Mice were treated as described in Fig. 1. (A) Sections of the liver tissue were analyzed immunohistochemically for the phosphorylated form of IKK. (B) The activation of NF-κB was measured by EMSA. All data are expressed as mean ± SEM (n = 8 for each group). *P<0.05 versus corresponding infected mice or the cyclodextrin (280 mg/kg) group.

IκBα acts as endogenous NF-κB inhibitor by keeping the proteins in the cytosolic localization. After IκBα is targeted for degradation by phosphorylation activated IKK, NF-κB translocates into the cell nucleus where it activates the expression of target genes. To address NF-κB signaling, we analyzed the phosphorylation of IKK as parameter of NF-κB activity. Indeed, our results show that phospho-IKK staining intensities in hepatic granuloma from BSE-CD -treated animals were clearly reduced in comparison to control mice (Fig. 2). We also investigated the effects of BSE-CD on NF-κB activation using an EMSA method. Fig. 2 shows that BSE-CD (280 mg/kg) suppressed the constitutive activation of NF-κB in the liver tissue.

### Early Phase BSE-CD Treatment Markedly Decreases MCP-1, TNF-α and VEGF mRNA Levels

**Figure 3:**
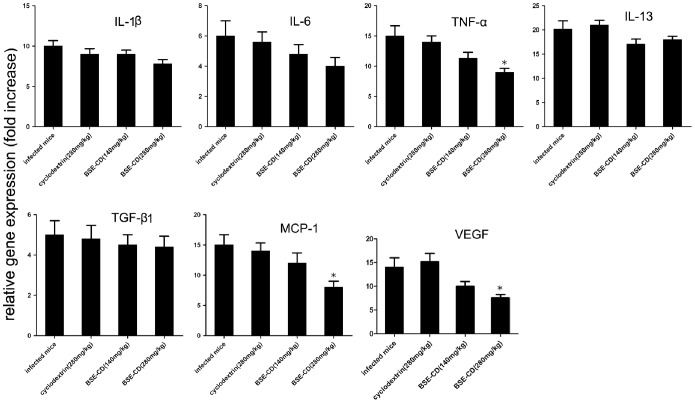
Effect of early phase BSE-CD treatment on cytokine mRNA expression in the liver of mice infected with *S. japonicum*. Mice were treated as described in Fig. 1. The mRNA levels were expressed relative to the levels in uninfected controls following normalization with β-actin. All data are expressed as mean ± SEM (n = 8 for each group). *P<0.05 versus corresponding infected mice or the cyclodextrin (280 mg/kg) group.

To explore the molecular mechanism underlying the anti-granuloma effect mediated by early phase BSE-CD treatment, we analyzed some gene mRNA levels in the liver of BSE-CD-treated mice compared with control mice. Our data show that MCP-1, TNF-α and VEGF mRNA expression as determined by real time PCR were dose-dependently decreased in the liver of BSE-CD (280 mg/kg)-treated mice (*P*<0.05). No significant differences in the expression of IL-1, IL-6, IL-13 and TGF-β were detected in the livers of the four groups (Fig. 3).

### Late Phase *BSE-CD* Treatment Reverses *S. japonicum* Egg-induced Liver Fibrosis

**Figure 4:**
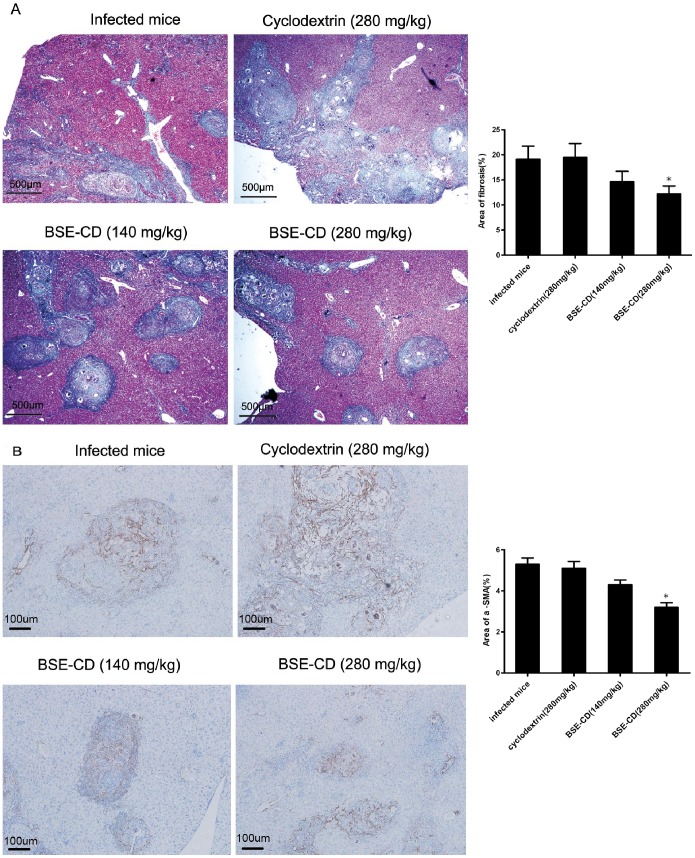
Effect of late phase BSE-CD treatment on hepatic fibrosis of liver tissue from mice that had been infected with *S. japonicum*. Mice were infected with 20±2 cercariae, Treatment with BSE-CD or placebo from week 7 until week 11. (A) Mice were killed, and liver tissues were fixed in formalin and stained with Masson trichrome or (B) anti-SMA antibody. Collagen deposition and the α-SMA positive areas were quantified as shown on the right hand panel, respectively. All data are expressed as mean ± SEM (n = 8 for each group). *P<0.05 versus corresponding infected mice or the cyclodextrin (280 mg/kg) group.

To investigate the effect of BSE-CD on *S. japonicum* egg-induced liver fibrosis, we assayed the effect of late phase BSE-CD treatment on pre-existent fibrosis, *i.e.* after fibrosis had already developed, We eliminated all worms in infected mice with praziquantel on week 6, and subsequently treated the mice with BSE-CD or placebo every day at 7 weeks (21 days of successive administration). Examination by Masson staining showed a significant decrease in the extent of fibrosis in the liver sections of BSE-CD (280 mg/kg)-treated mice compared with control mice (*P*<0.05) (Fig. 4A). The results also showed that more fibrous septa were formed in the model mice, and more collagen accumulated and deposited in the sinusoids and liver lobules. However, less fibrous septa and collagen were found in BSE-CD-treated mice (Fig. 4A). Furthermore, a significant decrease of α-SMA positive areas was also observed in BSE-CD (280 mg/kg)-treated mice compared with control mice (*P*<0.05) (Fig. 4B).

### Late Phase BSE-CD Treatment Markedly Decreases MCP-1, TNF-α and VEGF mRNA Levels

**Figure 5:**
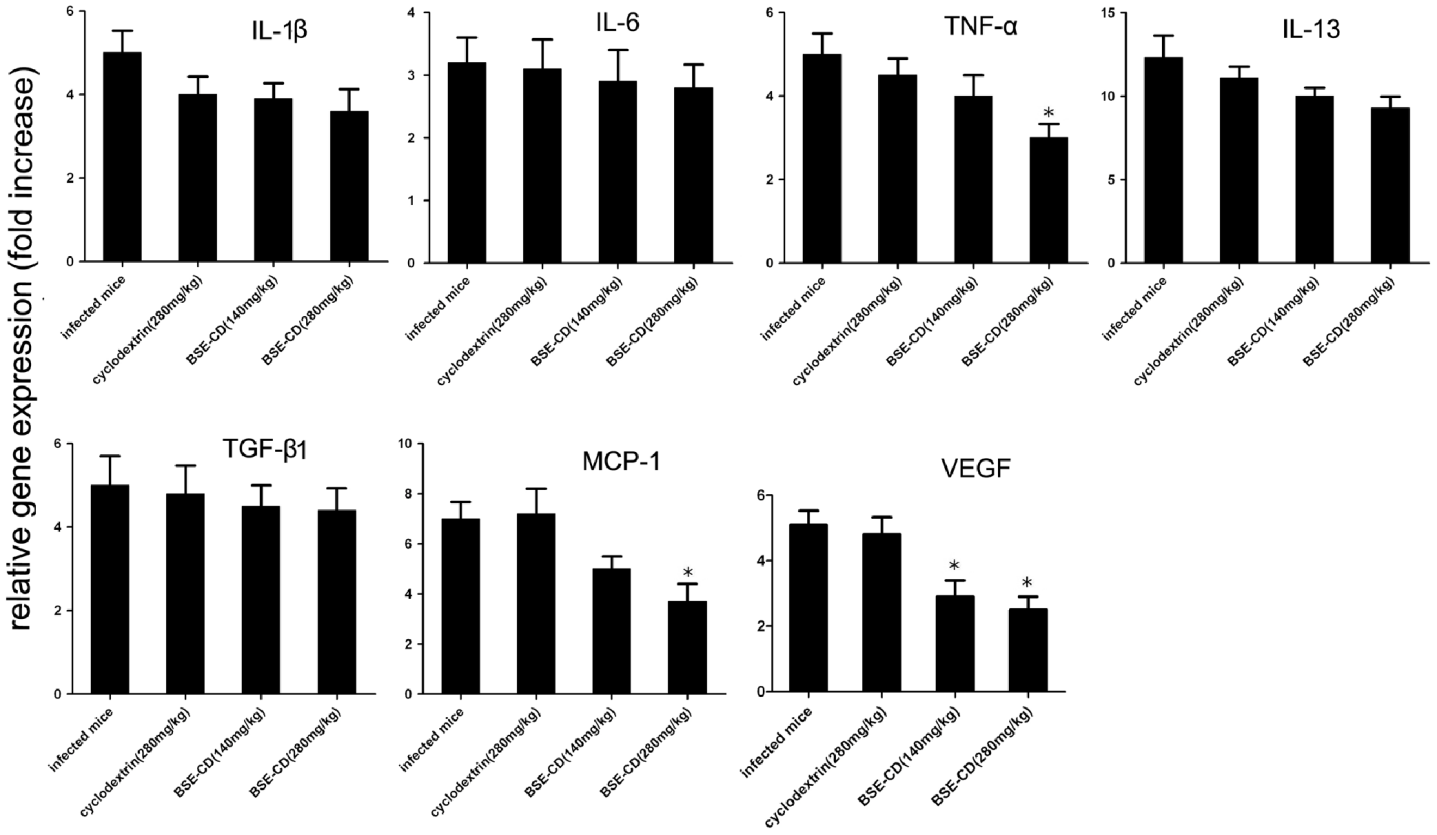
Effect of late phase BSE-CD treatment on cytokine mRNA expression in the liver tissue of mice. Mice were treated as described in Fig. 4. The mRNA levels were expressed relative to the levels in uninfected controls following normalization with β-actin. All data are expressed as mean ± SEM (n = 8 for each group). *P<0.05 versus corresponding infected mice or the cyclodextrin (280 mg/kg) group.

To explore the molecular mechanism underlying the anti-fibrotic effect mediated by the late phase BSE-CD treatment, we analyzed some gene mRNA levels in the liver of BSE-CD-treated mice compared with control mice. We found that the expression of MCP-1, TNF-α, and VEGF was decreased in liver tissue of BSE-CD (280 mg/kg)-treated mice as detected by real time PCR assay (*P*<0.05), whereas no difference was found regarding the expression of IL-1, IL-6, IL-13 and TGF-β in the livers of the four groups of mice (Fig. 5).

## Discussion

Although BSE is a contemporary phytomedicine, BA-containig remedies have already been used in traditional medicine for the treatment of various inflammatory diseases. The fact, that treatment with BSE and other BA-containing phytomedicines cause only minor side effects makes such preparations particularly appealing therapeutics [16]. The anti-inflammatory mechanism of BA-containing preparations such as BSE has been partly ascribed to their suppressive activity on lipid mediator biosynthesis encompassing leukotrienes and PGE_2_ [22]–[24]. More importantly, several BAs have been shown to interfere with NF-κB signaling leading to inhibition of proinflammatory NF-κB activation [25]–[28]. Similarly, Kumar confirmed acyl derivatives of BAs can inhibit NF-κB and STATs pathway [29]. Latella have demonstrated the extracts from *Boswellia serrata* and *Scutellaria baicalensis* prevented the development of colonic fibrosis in rats [18]. Ali found that a BA extract could attenuate pulmonary fibrosis induced by bleomycin and oxidative stress from gamma irradiation in rats [19]. We have confirmed that BSE-CD could attenuate *S. japonicum* egg-induced hepatic granuloma, which may be partly dependent on the down-regulation of some biochemical mediators [20]. Here, we wanted to study in greater detail the role of BAs in *S. japonicum* egg-induced liver granuloma and fibrosis. We found that early BSE-CD treatment significantly attenuated *S. japonicum* egg-induced granuloma and significantly improved hepatic gross appearance (Fig. 1). We also observed that late BSE-CD treatment significantly attenuated *S. japonicum* egg-induced fibrosis (Fig. 4).

In resting cells, the transcription factor NF-κB is bound to inhibitor of nuclear factor κB (IκB) proteins, keeping the transcription factor in a cytosolic localization. Upon stimulation by many factors, IκB kinases (IKK) become activated and mediate phosphorylation and degradation of the inhibitor. Subsequently, free NF-κB is translocating into the nucleus where it activates target gene expression [8]. In line with previous findings [25]–[29], our data show that phospho-IKK staining intensities in hepatic granuloma from BSE-CD (280 mg/kg) treated animals were clearly reduced in comparison to control mice (P<0.05). Besides EMSA data confirmed that *in vivo* treatment with BSE-CD (280 mg/kg) suppressed NF-κB signal transduction in the liver tissue (P<0.05) (Fig. 2).

The NF-κB signaling pathway modulates hepatic fibrogenesis predominantly by regulating stellate cell survival and hepatocyte injury, the primary effects of fibrogenic and inflammatory signals eliciting hepatic inflammation. Indeed, the activation of NF-κB seems to play a key role in the pathogenesis of liver fibrosis by regulating hepatocyte, HSC and Kupffer cell functions [10], [30]–[31]. Consistently, multiple studies have demonstrated inhibiton of hepatic fibrosis by targeting NF-κB signaling [32]–[33]. In line with those findings, BSE-CD could also significantly reduce the hepatic granuloma and fibrosis size in our study, which could at least be partly due to inhibiting the NF-κB signaling pathway.

The process of fibrosis formation from granular tissue in the liver is dependent on activation of stellate cells [34]. Activated HSCs are considered to be effector cells of liver fibrosis playing an important role in the granulomatous, fibrotic process induced by *S. japonicum* eggs [35]. Our data show that BSE-CD could markedly reduce HSC activation (Fig. 4), which is expected to contribute to the suppression of hepatic fibrosis formation.

Vascular endothelial growth factor (VEGF) contributes to the regulation of vasculogenesis, and induces angiogenesis and endothelial cell proliferation [36]. Some studies have linked VEGF with harmful effects in the state of hepatic granuloma and fibrosis and cirrhosis in patients and experimental animal models [37]–[38]. Consistently, application of an angiogenic inhibitorin an experimental murine model of *S. mansoni* resulted in reduced injury characterized by attenuated hepatic granuloma formation and fibrosis [15]. MCP-1can trigger migration of monocytes into the hepatic granuloma. To that effect, the expression profile of MCP-1 has close correlation with granuloma diameter. It has been reported that MCP-1 might play a key role in initiating and maintaining the immune response to tissue egg deposition, injection of anti-MCP-1 antibodies could inhibit granuloma formation [13]. TNF-α is a cytokine taking part in many processes, including inflammation, proliferation and programmed cell death. Studies *in vitro* and in experimental models *in vivo* have demonstrated that TNF-α plays a role in granuloma formation and hepatic fibrosis [39]. In fact, TNF-α seems to play a central role in promoting periportal fibrosis during *S. mansoni* infection and was found to be elevated in peripheral blood eosinophils from chronic Schistosoma mansoni-infected patients [40].

Multiple studies have demonstrated that BA treatment significantly decreases VEGF [25], [28], [41]–[42], MCP-1 [25], [28] and TNF-α [28], [40], [43] in different animal model such as in LPS-challenged ApoE mice [25], [28], in a transgenic model of psoriasis [44], in an orthotopic nude mouse model of PaCa [41] and in a murine sponge model [43]. Our results show that the early and late treatment of mice with *S. japonicum* infection by BSE-CD also significantly reduces VEGF, TNF-α and MCP-1 levels; the genes of those proteins were reported to be under the control of NF-κB (Fig. 3, Fig. 5). The attenuating effect of BSE-CD on liver granuloma and fibrosis was accompanied by decreased MCP-1, TNF-α, and VEGF levels expression, which may contribute to the inhibition of macrophage recruitment, and to the reduction of peri-ovular inflammatory responses and vasculogenesis.

Both IL-13 and TGF-β1 are involved in the activation of “resting” HSCs and they are known to play an important role in promoting hepatic fibrosis in schistosomiasis [35], [45]. There have been reports that BSE treatment could downregulate the expression of transforming growth factor (TGF-β 1) in vivo [19]. However, we found BSE treatment had little effect on TGF-β 1 and IL-13 mRNA expression in the liver tissue (Fig. 3, Fig. 5). Thus, BSE-CD treatment attenuates *S. japonicum* egg-induced hepatic fibrosis independent from a decreased expression of IL-13 and TGF-β1. We also observed that the expression level of IL-1β and IL-6 tended to decrease in the BSE-CD-treatment groups. However, there was no significant difference in the expression of IL-1β and IL-6 between the four group mice (Fig. 3, Fig. 5).

In conclusion, our data show that BSE-CD treatment can attenuate *S. japonicum* egg-induced hepatic granulomas and fibrosis, which might be partly due to the suppression of the NF-κB pathway and a decreased expression of VEGF, TNF-α and MCP-1. Suppression of HSC activation may also play a role in the therapeutic effect of BSE-CD. This study explores BSE-CD in treatment of *S. japonicum* egg-induced hepatic granuloma and fibrosis as well as its mechanism. Our findings may provide the theoretical and practical basis for a novel treatment of schistosomal hepatic granuloma and fibrosis.

## Supporting Information

Table S1 Sequences of primers used in this study.
(DOCX)

Table S2 Parasitological measurements in infected mice treated with BSE-CD or cyclodextrin in early phase. Mice were treated as described in Fig. 1, and then we measured worm pairs, total worms, and total parasite eggs in the livers. Worm pairs, total worms, and total parasite eggs in the livers were similar between BSE-CD treatment groups and the control groups(*P*>0.05). All data are expressed as mean ± SEM (n = 8 for each group).
(DOCX)
