# Supplementary material for: A Boswellic Acid-Containing Extract Ameliorates Schistosomiasis Liver Granuloma and Fibrosis through Regulating NF-κB Signaling in Mice
Source: PLoS One. 2014 Jun 18;9(6):e100129. doi: 10.1371/journal.pone.0100129 (PMC4062494; doi:10.1371/journal.pone.0100129)
Supplement: Table S1 — Sequences of primers used in this study. (DOCX) [file pone.0100129.s001.docx]

| Genes | Forward Primer(5' to 3') | Reverse Primer(5' to 3') |
| --- | --- | --- |
| IL-1β  IL-6  TNF-α  TGFβ1  IL-13  MCP-1  VEGF | CTGAACTCAACTGTGAAATGC  ACCACGGCCTTCCCTACTT  ACTGGCAGAAGAGGCACTC  ACAATTCCTGGCGTTACCTT  CCTGGCTCTTGCTTGCCTT  TTAAAAACCTGGATCGGAACCAA  GTAACGATGAAGCCCTGGAGTG | TGATGTGCTGCTGCGAGA  CACAACTCTTTTCTCATTTCCAC  CTGGCACCACTAGTTGGTTG  AGCCCTGTATTCCGTCTCC  GGTCTTGTGTGATGTTGCTCA  GCATTAGCTTCAGATTTACGGGT  CATCTGCTGTGCTGTAGGAAGC |
| β-actin | AGAGGGAAATCGTGCGTGAC | CAATAGTGATGACCTGGCCGT |
