# Supplementary material for: A Boswellic Acid-Containing Extract Ameliorates Schistosomiasis Liver Granuloma and Fibrosis through Regulating NF-κB Signaling in Mice
Source: PLoS One. 2014 Jun 18;9(6):e100129. doi: 10.1371/journal.pone.0100129 (PMC4062494; doi:10.1371/journal.pone.0100129)
Supplement: Table S2 — Parasitological measurements in infected mice treated with BSE-CD or cyclodextrin in early phase. Mice were treated as described in Fig. 1, and then we measured worm pairs, total worms, and total parasite eggs in the livers. Worm pairs, total worms, and total parasite eggs in the livers were similar between BSE-CD treatment groups and the control groups(P>0.05). All data are expressed as mean ± SEM (n = 8 for each group). (DOCX) [file pone.0100129.s002.docx]

| Group | No | Total worms | Worm pairs | Total liver eggs (×10^3^) |
| --- | --- | --- | --- | --- |
| Infected mice | 8 | 13.9 ± 1.35 | 5.7± 0.52 | 40.02 ± 5.11 |
| Cyclodextrin(280mg/kg) | 8 | 14.5 ± 1.23 | 6.0± 0.32 | 37.35 ± 4.91 |
| BSE-CD (140 mg/kg) | 8 | 13.4 ± 1.65 | 5.8± 0.61 | 38.23 ± 3.39 |
| BSE-CD (280 mg/kg) | 8 | 14.0 ± 1.84 | 5.9± 0.42 | 39.73 ± 4.31 |
